# Lysine *α*-ketoglutarate reductase as a therapeutic target for saccharopine pathway related diseases

**DOI:** 10.3389/fnmol.2025.1695490

**Published:** 2025-10-21

**Authors:** Gabriel Vieira Valderrama, Gabriela Alves Moreira, Paulo Arruda

**Affiliations:** ^1^Centro de Química Medicinal, Universidade Estadual de Campinas, Campinas, Brazil; ^2^Centro de Biologia Molecular e Engenharia Genética, Universidade Estadual de Campinas, Campinas, Brazil; ^3^Departamento de Genética, Evolução, Microbiologia e Imunologia e Evolução, Instituto de Biologia, Universidade Estadual de Campinas, Campinas, Brazil

**Keywords:** saccharopine pathway, lysine catabolism, rare genetic diseases, pyridoxin dependent epilepsy, glutaric aciduria, saccharopinuria

## Abstract

The saccharopine pathway (SacPath) and the pipecolate pathway (PipPath) catabolize lysine to α-aminoadipate. Although the PipPath has been highlighted as the prominent route operating in the brain, recent work has demonstrated that the SacPath plays a major role in lysine catabolism in the brain. The first two enzymatic steps of the SacPath involve the bifunctional enzyme α-aminoadipate semialdehyde synthase (AASS) harboring the lysine-ketoglutarate reductase (LKR) and the saccharopine dehydrogenase (SDH) domains that convert lysine to α-aminoadipate semialdehyde. Thereafter, the semialdehyde is converted to α-aminoadipate by α-aminoadipate semialdehyde dehydrogenase (AASADH). Mutations abolishing the enzymatic activities of LKR, SDH, and AASADH lead to the genetic diseases hyperlysinemia type I and II, and pyridoxine-dependent epilepsy (PDE), respectively. Hyperlysinemia type I accumulates lysine and causes a benign phenotype without clinical significance. Hyperlysinemia type II accumulates saccharopine, which leads to neuronal disorders and intellectual disability. PDE accumulates α-aminoadipate semialdehyde and its cyclic isomer piperideine-6-carboxylate, which binds pyridoxal 5′-phosphate, disturbs synapses, and causes seizures along with developmental disorders. Another genetic disease, glutaric aciduria type I (GA1), localizes just downstream of the SacPath and is caused by mutations abolishing the enzymatic activity of glutaryl-CoA dehydrogenase (GCDH). GA1 accumulates glutarate and 3-hydroxyglutarate, which are neurotoxic molecules that cause irreversible brain damage. Downregulation of LKR has been shown to reduce the metabolic flux through SacPath and alleviate PDE and GA1 symptoms. This review discusses the role of SacPath and its enzymes as potential targets for developing drugs to treat PDE and GA1, as well as other diseases.

## 1 Introduction

The understanding of the impact of intermediary metabolite perturbations on cellular biology is essential to accurately identify potential targets for drug development toward disease treatment. Many diseases arise from genetic mutations abolishing gene/protein function or alteration of gene expression, leading to loss of cellular homeostasis (Shovlin and Aldred, 2025). Rare genetic diseases are essentially caused by monogenic mutations that, upon homozygosity, lead to overaccumulation of intermediary metabolites, causing severe phenotypes limiting the life quality and span (Barroso and McCarthy, 2019). Loss-of-function mutations in genes encoding enzymes of amino acid metabolism have long been associated with mild and severe diseases (Aliu et al., 2018; Yahyaoui and Pérez-Frías, 2019). The lysine catabolic pathways are highly relevant because of the multiple neuronal diseases associated with it (Chang, 2023). One of the best-characterized cases diseases associated with lysine catabolism is pyridoxine-dependent epilepsy (PDE), caused by the loss-of-function mutations of the enzyme *α*-aminoadipate semialdehyde dehydrogenase (AASADH) (also known as antiquitin, and aldehyde dehydrogenase 7A1, ALDH7A1) (Mills et al., 2006). PDE patients accumulate *α*-aminoadipate 6-semialdehyde (AASA) and its cyclic form, piperideine 6-carboxylate (P6C), and pipecolic acid (PIP) in plasma and cerebrospinal fluid (Plecko et al., 2005; Bok et al., 2007; Mills et al., 2010; Stockler et al., 2011). Excess of AASA/P6C can condense with pyridoxal 5′-phosphate (PLP), depleting this coenzyme in the neurons, disturbing the synapses, and causing seizures (Mills et al., 2006; Stockler et al., 2011). Loss of AASADH activity may affect other cellular functions, as the enzyme has been shown to play crucial roles as an aldehyde scavenger, thus alleviating oxidative stress (Brocker et al., 2011; Brocker et al., 2010). The role of AASADH as an aldehyde scavenger may have implications for other diseases. For example, the enzyme has been shown to be strongly upregulated, leading to overaccumulation of a-aminoadipate (AAA) in glioblastoma (Rosi et al., 2015), prostate cancer (Van Den Hoogen et al., 2011), lung cancer (Giacalone et al., 2013), adenocarcinoma (Lee et al., 2021), and diabetes (Wang et al., 2013; Koos and Gornbein, 2021). In almost all these cases, overaccumulation of AAA is correlated with disease aggressiveness.

Regarding neuronal diseases, two metabolic pathways have been proposed for lysine to AASA catabolic flux in the brain: the saccharopine pathway (SacPath), and the pipecolate pathway (PipPath). Early data provided evidence for the operation of the SacPath in the brain (Papes et al., 1999). However, this has been contested, as although the first two enzymes of the SacPath lysine-ketoglutarate reductase (LKR) and saccharopine dehydrogenase (SDH) have been detected in the central nervous system, this was attributed to extracellular activity in neuronal cells (Papes et al., 1999; Hallen et al., 2013). However, recent data clearly show that the lysine to *α*-aminoadipate 6-semialdehyde catabolic flux occurs in the brain mainly through the SacPath (Pena et al., 2017; Crowther et al., 2019). These findings bring an exceptional opportunity to develop drugs targeting the first enzymatic steps of SacPath and thus develop a treatment for PDE. In addition, other rare genetic diseases such as saccharopinuria type II and the downstream glutaric aciduria (GA1) could also benefit from a drug targeting the first enzymatic steps of the SacPath (Pena et al., 2017; Pena et al., 2016). In addition, such a drug for PDE may be helpful to downregulate the AASADH activity, with a potentially beneficial role in reducing tumor progression (Lee et al., 2021). This review provides an update and new insights into the role of SacPath in neuronal and other diseases.

## 2 Lysine catabolic pathways in mammals

**Figure 1:**
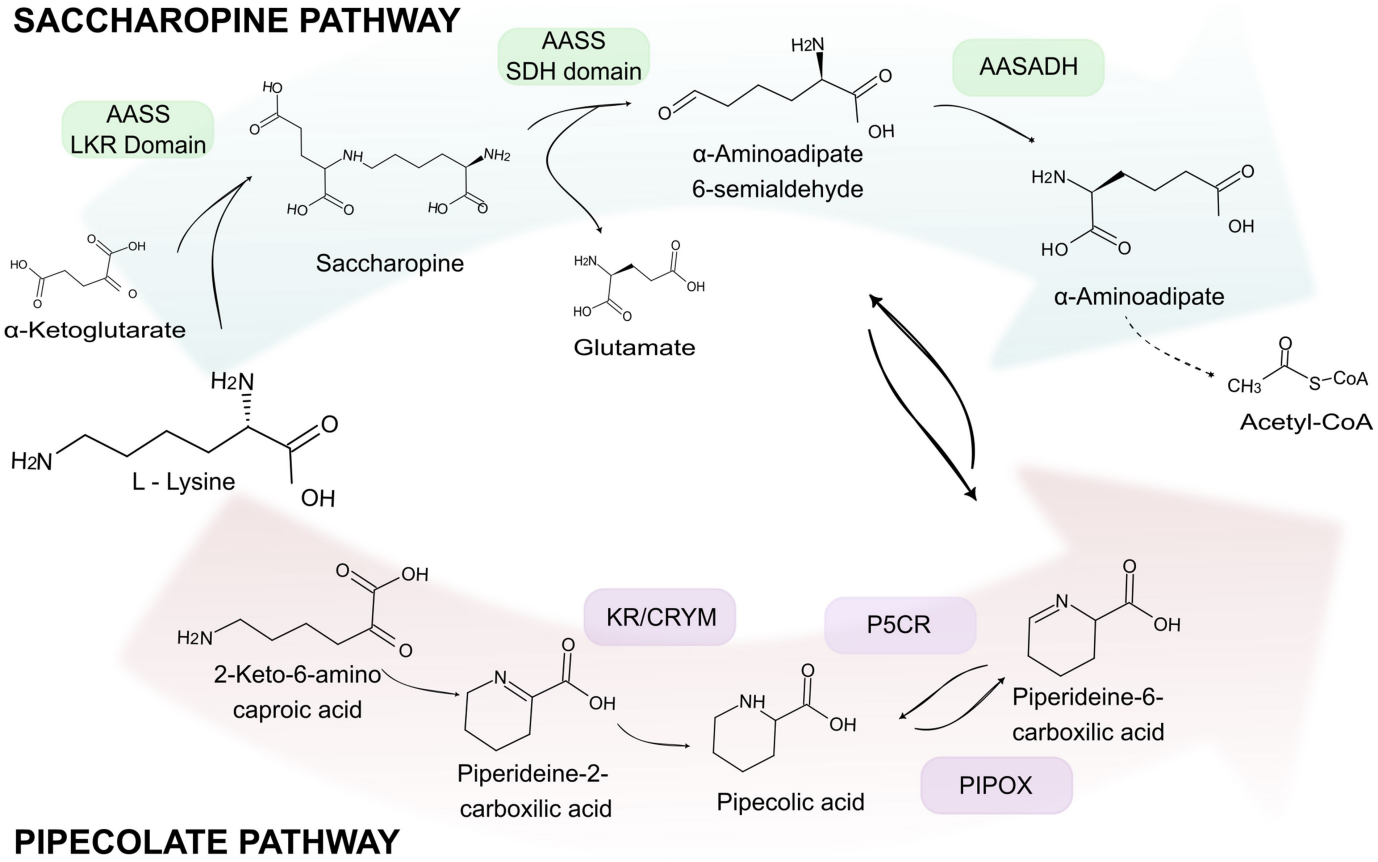
Rare genetic diseases caused by loss-of-function mutations in the enzymes of the SacPath. The SacPath, which is located in the mitochondria, is associated with four rare genetic disorders. (1) Hyperlysinemia Type I, caused by mutations in the LKR domain of the AASS gene, leads to the accumulation of lysine. It typically does not produce symptoms and is considered a benign metabolic variant. (2) Hyperlysinemia Type II or Saccharopinuria caused by mutations in the SDH domain of AASS leads to the accumulation of saccharopine. This accumulation is toxic to mitochondria and can lead to neuropathological consequences. (3) Pyridoxin-dependent epilepsy, caused by mutations in the gene encoding AASADH. This condition leads to an accumulation of α-aminoadipate semialdehyde and its cyclic form, piperideine-6-carboxylate. These compounds bind to pyridoxal 5′-phosphate (PLP), depleting this crucial cofactor in the brain and causing epilepsy episodes, along with neurodevelopmental delays and other neuropathological symptoms. (4) Glutaric Aciduria, caused by mutations in the gene encoding the enzyme GCDH, leading to the accumulation of glutaryl-CoA (G-CoA). The accumulation of this neurotoxic compound causes irreversible brain damage in infancy, resulting in severe neurological issues.

Lysine catabolism in mammals occurs through two main pathways: the PipPath and SacPath. The PipPath begins with the deamination of lysine by an unidentified enzyme, resulting in the formation of 2-Keto-6-amino caproic acid (KAC). KAC then spontaneously condenses through imine bond formation to produce piperideine-2-carboxylic acid (P2C). This compound is subsequently reduced to PIP by Ketimine reductase (KR/CRYM). Then, PIP is oxidized via the action of pipecolic acid and sarcosine oxidase (PIPOX), leading to the formation of piperideine-6-carboxylic acid (P6C). This reaction is reversible and can also be catalyzed by pyrroline-5-carboxylate reductase (P5CR) (Figure 1) (Chang, 1978; Murthy and Janardanasarma, 1999; Struys et al., 2014). The SacPath, located in the mitochondria, begins with the formation of saccharopine through the condensation of lysine and *α*-ketoglutaric acid by LKR, the first enzymatic domain of the bifunctional enzyme aminoadipate semialdehyde synthase (AASS). Subsequently, the second enzymatic domain of AASS, SDH, hydrolyzes saccharopine into *α*-aminoadipate 6-semialdehyde and glutamate. α-Aminoadipate 6-semialdehyde is then oxidized into α-aminoadipate (AAA) by the action of AASADH (Figure 1). A connection between the pathways is established via the spontaneous and reversible cyclization of AASA, leading to the formation of P6C (see Figure 1) (Pena et al., 2017; Pena et al., 2016; Struys and Jakobs, 2010).

Historically, it was believed that lysine degradation in the brain primarily occurred through the PipPath. However, over the past two decades, substantial evidence has emerged, demonstrating that the SacPath is, in fact, the primary route of lysine catabolism not only liver and kidney, but also in the brain (Papes et al., 1999; Hallen et al., 2013; Pena et al., 2017; Crowther et al., 2019; Pena et al., 2016; Chang, 1978; Struys and Jakobs, 2010). Key studies using isotopic tracing with L-[*α*-15 N] and L-[*ε*-15 N]Lysine in AASADH-deficient human fibroblasts demonstrated that L-[α-15 N] Lysine was metabolized into [α-15 N] saccharopine, [α-15 N] AASA, [α-15 N] P6C, and [α-15 N] PIP, highlighting the significance of the SacPath in lysine catabolism in cellular models (Struys and Jakobs, 2010). Additionally, recombinant human P5CR was found to convert P6C solely into PIP, connecting SacPath-derived intermediates back to PipPath metabolites (Struys et al., 2014). Studies using mouse models have provided valuable insights into the tissue-specific catabolism of lysine. Detailed time-course experiments showed that 2 h after an intraperitoneal injection of lysine, there is a peak in the levels of catabolites in the liver, kidney, and brain. Furthermore, the SacPath was identified as the primary pathway for the degradation of lysine (Pena et al., 2016). The predominance of SacPath in lysine catabolism was also demonstrated in human neuronal progenitor cells and fibroblasts (Crowther et al., 2019). Thus, the SacPath is central to lysine catabolism and has significant implications for brain homeostasis and in numerous neurological disorders associated with the accumulation of SacPath intermediates (Chang, 2023; Guo et al., 2022).

## 3 Rare genetic diseases associated with lysine catabolism

**Figure 2:**
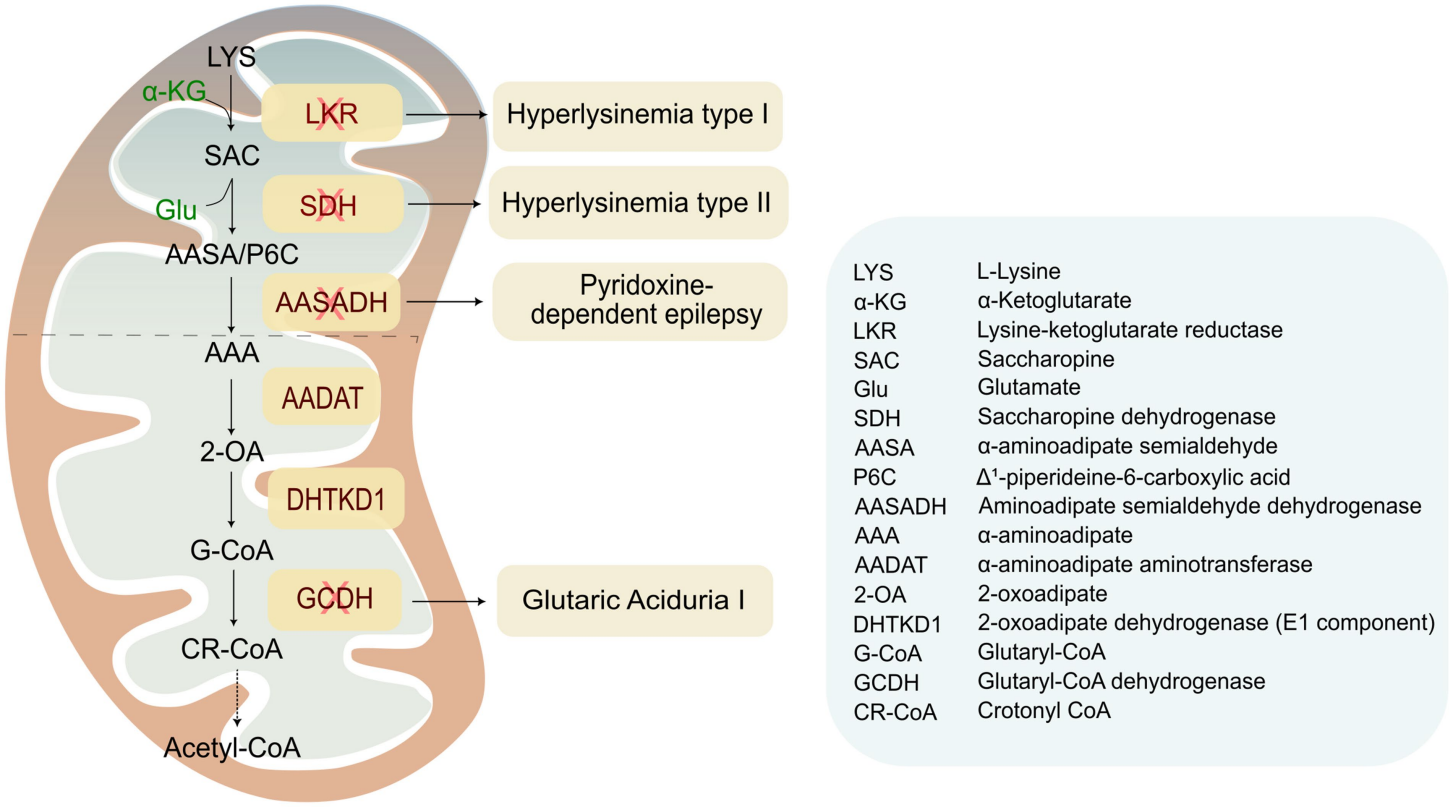
Lysine catabolic pathways. Lysine catabolism occurs through two primary pathways: the saccharopine pathway (SacPath) and the pipecolate pathway (PipPath). The first two steps of the SacPath are catalyzed by the bifunctional enzyme AASS that comprises the LKR and the SDH domains. The LKR domain catalyzes the condensation of lysine and a-ketoglutarate into saccharopine. The SDH domain hydrolyses saccharopine into α-aminoadipic semialdehyde and glutamate. Subsequently, aminoadipic semialdehyde is oxidized to form α-aminoadipate, which is further converted into 2-oxoaminoadipate, glutaryl-CoA, and crotonyl-CoA. In the pipecolate pathway, lysine is converted into pipecolic acid through several intermediates, specifically 2-keto-6-amino caproic acid and piperideine-2-carboxylic acid. This conversion involves the reduction by the enzymes KR/CRYM, followed by the reversible oxidation of PIP to P6C, which is catalyzed by either PIPOX or P5CR.

At least four rare genetic neurological diseases have been linked to lysine catabolism. These include the ultra-rare hyperlysinemia types I and II, as well as PDE and GA1. These conditions are caused by mutations in the LKR, SDH, AASADH, and GCDH genes, respectively (Figure 2).

### 3.1 Hyperlysinemia type I and II

Lysine accumulation, known as hyperlysinemia type I, is caused by an impairment in the activity of the LKR domain of the AASS gene. This condition is characterized as a benign biochemical phenotype; individuals with type I hyperlysinemia do not exhibit any symptoms, although further confirmation is still required this metabolic disruption appears to have no adverse health effects (Dancis et al., 1983; Houten et al., 2013). The same cannot be said in the case of accumulation of saccharopine, as a mutation in the SDH domain of AASS leads to hyperlysinemia type II or saccharopinuria, a mild symptomatic disease that, in some cases, can lead to detrimental neurological dysfunction, retardation, and brain malfunction (Houten et al., 2013; Carson et al., 1968). It remains necessary to confirm whether type II consistently manifests as a harmful condition, but in animal models, saccharopinuria was associated with mitochondrial dysfunction, such as abnormality in morphology, impairment in fission, and ER-dependent mitochondria tubulation, leading to liver diseases, neurodevelopmental delay, and premature death. In LKR and SDH double mutants, the accumulation of saccharopine was not detected, and the high level of lysine did not affect mitochondrial function (Zhou et al., 2019).

### 3.2 Pyridoxine dependent epilepsy

Pyridoxine-dependent epilepsy (PDE) is a hallmark of diseases associated with the SacPath. It is caused by homozygous mutations in the AASADH gene, leading to the overaccumulation of AASA and P6C. PDE was first described in 1954 and is characterized by intractable neonatal or infantile seizures that are resistant to conventional antiepileptic drugs but respond well to high doses of pyridoxine (Hunt et al., 1954; Stockler et al., 2011; Clayton, 2006; Goto et al., 2001). P6C undergoes a spontaneous Knoevenagel condensation with pyridoxal-5′-phosphate (PLP), the active form of vitamin B6. This reaction inactivates PLP, which is an essential cofactor for enzymes involved in the synthesis and degradation of amino acids and neurotransmitters. PLP is crucial for dopaminergic, serotonergic, glutamatergic, and GABAergic neurotransmission (Mills et al., 2006; Clayton, 2006; Percudani and Peracchi, 2003). The lack of this co-factor affects the activity of the PLP-dependent enzyme glutamate decarboxylase, resulting in lower GABA levels, which has been previously suggested as a mechanism for pyridoxine-dependent seizures (Goto et al., 2001). Eighty pathogenic allelic variants have been identified for the PDE within the 18 exons of the *ALDH7A1*. The missense mutation E399Q accounts for approximately 30% of all reported alleles. This specific residue is known to play a crucial role in forming a hydrogen bond with the nicotinamide group of NAD+, which leads to a loss of AASADH activity (Coughlin et al., 2019; Bennett et al., 2009). Genotype–phenotype correlations for PDE suggest three distinct groups of clinical phenotypes: (1) the first group includes patients who achieve complete seizure control with pyridoxine and demonstrate normal developmental, (2) the second group consists of patients whose seizures are managed effectively with pyridoxine supplementation, but they experience developmental delays, (3) the third group comprises patients who continue to have seizures despite pyridoxine treatment and show developmental impairments. Mutations resulting in residual AASADH activity are more commonly observed in the first group. However, these correlations are not always consistent, as patients with homozygous mutations E399Q have been reported in both the first and third groups (Bennett et al., 2009; Scharer et al., 2010).

The lifelong treatment for PDE includes high-dose supplementation of pyridoxal, which controls seizures by reducing PLP depletion. However, over 75% of patients continue to experience post-natal developmental delays (Van Karnebeek et al., 2016; Basura et al., 2009). A triple therapy approach that includes a lysine-restricted diet and arginine supplementation has demonstrated additional benefits for patients by lowering lysine levels in neuronal cells through decreased transport availability and competition with arginine (Yuzyuk et al., 2016; Coughlin et al., 2015; Johal et al., 2024). PDE is still not included in standard newborn screening protocols, although recent studies have highlighted its feasibility in dried blood spot testing (Pauly et al., 2025). The accumulation of AASA/P6C is detrimental to general cellular homeostasis, exerting additional, PLP-independent toxicity (Brocker et al., 2011). Studies using zebrafish and mouse models lacking *Aldh7a1* shows that these compounds induce endoplasmic reticulum stress responses and disrupt redox balance, as demonstrated by increased ROS levels and changes in oxidative stress marker expression (Pena et al., 2017). Additionally, *Aldh7a1*-deficient mice exhibit impaired adult hippocampal neurogenesis due to the accumulation of AASA/P6C, which inhibits the proliferation and differentiation of neural stem cells (NSCs). This impairment is also linked to disruptions in *de novo* pyrimidine biosynthesis. Supplementation with pyrimidine precursors such as uridine, thymidine, and cytidine can reverse both neurogenic and cognitive deficits. Moreover, mice with astrocyte-specific deletion of *Aldh7a1* show changes in dendritic spine composition, a reduction in synapsin-positive puncta, and a decreased frequency of both excitatory and inhibitory postsynaptic currents, further supporting the notion of widespread synaptic imbalance (Yan et al., 2024; Al-Shekaili et al., 2020).

### 3.3 Glutaric aciduria type I

Mutations in the GCDH, an enzyme localized just downstream of the SacPath (Figure 2), lead to GA1 disease, also known as Glutaric Aciduria type I (Goodman et al., 1977). This enzyme is essential for the metabolism of lysine, hydroxylysine, and tryptophan, as it catalyzes the conversion of glutaryl-CoA into crotonyl-CoA (Yuan et al., 2023). A deficiency in GCDH results in the accumulation of glutaryl-CoA and its related metabolites, glutaric acid (GA) and 3-hydroxyglutaric acid (3-OH-GA), both of which are toxic to neurons (Leandro et al., 2020). GA1 was first described by Goodman et al. in 1975 in siblings who exhibited neurodegenerative symptoms, including opisthotonos, dystonia, and athetoid posturing, beginning around 6 months of age. Additional clinical features may include macrocephaly at birth or shortly thereafter, developmental delays or regressions, generalized rigidity, seizures, and dyskinetic movements, such as oral and facial dyskinesia (Goodman et al., 1977). Many patients experience acute encephalopathic crises during catabolic states triggered by febrile illnesses, vaccinations, surgical procedures, or infections, particularly in the first 6 years of life if left untreated (Sahoo et al., 2017; Strauss et al., 2003). Newborn screening (NBS), which detects glutarylcarnitine (C5DC) in dried blood spots, enables presymptomatic diagnosis and timely intervention (Boy et al., 2023) Recommended therapy includes lysine-restricted diet with lysine-free, arginine-enriched amino acid mixtures until age six, lifelong carnitine supplementation, and emergency protocols during catabolic stress; after 6 years, the diet can be relaxed to controlled protein intake (Boy et al., 2023; Schmidt et al., 2020). There are several pathogenic variants of GCDH, many of which result in elevated levels of urinary GA and 3-OH-GA (Kölker et al., 2006; Heringer et al., 2010).

The role of the saccharopine pathway in GA1 pathophysiology has been debated. An earlier study using *Gcdh^−^/^−^* mice indicated that the PIP pathway is dominant in the brain, as evidenced by the presence of PIPOX and the lack of AASS activity (Sauer et al., 2011). Subsequent studies using a mouse model of L-2-hydroxyglutaric aciduria, which is clinically and metabolically similar to GA1, revealed that AASS is expressed in the brain (Rzem et al., 2015). Additionally, this study showed that L-2-hydroxyglutaric acid (HGA) inhibits the LKR domain of AASS, indicating a physiological feedback inhibition mechanism (Rzem et al., 2015). Recently, double knockout models for AASS and GCDH provided clear evidence that the SacPath is the primary source of glutaric acid production in neuronal tissue, as the accumulation of glutaric acid was significantly reduced in these models (Leandro et al., 2020).

### 3.4 Other diseases associated with the SacPath

Although rare monogenic disorders, such as hyperlysinemia, PDE, and GA1, are the most well-characterized conditions involving the SacPath, increasing evidence suggests its broader significance in complex multifactorial diseases. SacPath is activated under multiple stress conditions, like osmotic, oxidative, or nutritional leading to lysine catabolism via a cascade of intermediates, including AAA, AASA/P6C, and pipecolic acid (PIP). These molecules act as osmoprotectants, redox modulators, or immune effectors in stressed cells (Brocker et al., 2011; Brocker et al., 2010; Wang et al., 2013; Pérez-Arellano et al., 2010). Importantly, a lysine overload, whether originating from within the body or from external sources, triggers the transcription and translation of the SacPath pathway. This indicates that the pathway is part of a metabolic adaptation program in response to stress (Kiyota et al., 2015). In cancer, the activation of SacPath has been reported in several tumor types, particularly due to the overexpression of AASADH. This has been observed in models of prostate and ovarian cancer, as well as glioblastoma. Elevated levels of AASADH seem to enhance tumor aggressiveness by maintaining redox balance and clearing cytotoxic intermediates (Rosi et al., 2015; Van Den Hoogen et al., 2011; Saw et al., 2012). These findings indicate that the SacPath not only buffers metabolic stress in normal cells but may also provide a survival advantage for cancer cells, making its enzymes potential therapeutic targets.

In gestational diabetes mellitus (GDM), a metabolomic study identified elevated urinary saccharopine as the second most predictive biomarker after oxidized glutathione (Koos and Gornbein, 2021). This suggests SacPath activation may play a role in managing oxidative stress caused by maternal hyperglycemia. Given that both saccharopine and AASA have mitochondrial toxicity, this raises the hypothesis that SacPath may contribute to mitochondrial dysfunctions observed in GDM and possibly in type 2 diabetes as well (Zhou et al., 2019). Furthermore, metabolites derived from SacPath, especially PIP, have been found to contribute to inflammatory and redox regulation. They do this by promoting the production of nitric oxide (NO) and reactive oxygen species (ROS), which links this pathway to immune system activation and oxidative signaling (Wang et al., 2013; Lenk et al., 2019). Although the connection between SacPath and neurodegenerative conditions is still speculative, mitochondrial disruption, redox imbalance, and aldehyde accumulation, known consequences of SacPath dysregulation, are central features of diseases like Alzheimer’s and Parkinson’s (Anandhan et al., 2017). This positions SacPath as a potential player in a wider range of diseases, extending well beyond rare inborn errors of metabolism.

## 4 LKR as a target for drug development to treat human diseases

Substrate reduction therapy (SRT) is a promising treatment for amino acid metabolic disorders that involve the accumulation of toxic metabolites (Yue et al., 2019). The strength of this strategy lies in its capacity to reduce substrate flow upstream of the metabolic pathway, thereby decreasing downstream pathogenic processes and alleviating cellular stress as well as potential organ damage (Leandro et al., 2020). In this context AASS must be considered as a promising target for SRT in diseases caused by mutations affecting SacPath. Consisting evidences (Leandro et al., 2020; Liang et al., 2025; Minenkova et al., 2021) suggests that inhibiting the LKR domain of AASS could be a viable approach for targeting disorders involving SacPath, such as PDE and GA1. The double knockout of AASADH and LKR demonstrates a favorable profile, showing no adverse neurological effects, along with normalized neurodevelopmental outcomes and seizure phenotypes (Liang et al., 2025; Leandro and Houten, 2019). However, targeting AASS for drug development toward treatment of the SacPath-associated diseases should consider potential adverse functional outcomes due to its dual metabolic role as a bifunctional enzyme and its possible role in alleviating cellular stress. Inhibiting the LKR domain of AASS would reduce the flow of lysine to AAA, which would reduce the accumulation of toxic intermediates while increasing the lysine levels. Elevated levels of lysine do not negatively affect amino acid transport or brain function, thus assuring a benefic functional outcome (Pena et al., 2017; Zhou et al., 2019). In addition, inhibiting the LKR domain of AASS would help reduce the accumulation of toxic levels of glutaric acid and its derivatives, thereby contributing to the therapeutic treatment of GA1 (Leandro et al., 2020; Barzi et al., 2023; Segur-Bailach et al., 2025). Thus, developing a small molecule targeting LKR could lead to the creation of a single drug effective for two significant genetic rare diseases. In contrast, we should avoid developing a drug targeting the SDH domain of AASS, as this could lead to the accumulation of saccharopine, which is linked to mitochondrial dysfunction and impaired electron transport chain activity (Zhou et al., 2019; Liang et al., 2025; Minenkova et al., 2021).

Despite the significant potential of developing an LKR inhibitor as a treatment for the rare SacPath genetic disease, we must enhance our understanding of the physiological consequences of reducing the metabolic flow from lysine to AAA. This includes investigating the role of PIP and its derivatives in alleviating stress conditions and boosting the immune response. For instance, the expression of SacPath has been linked to cancer and diabetes, where the pathway is often upregulated (Wang et al., 2013; Koos and Gornbein, 2021). We do not yet know if the upregulation of SacPath benefits tumor progression, but upregulation of the lysine to AAA flux may increase the AASADH activity and its role as an aldehyde scavenger (Brocker et al., 2011; Brocker et al., 2010) may benefit the tumor cells. If this is the case, LKR inhibition may suppress lysine to AAA flux, prevent AASADH upregulation and its effect as aldehyde scavenger ultimately inhibiting cellular proliferation. Alternatively, targeting downstream AASADH or P5CR could elevate toxic intermediates, potentially impairing tumor progression (Brocker et al., 2010; Tanner et al., 2018).

The recent elucidation of the crystal structure of the human LKR domain offers a promising foundation for rational drug design, with further structural analysis of SacPath enzymes expected to expedite the development of effective therapeutics (Leandro et al., 2022). Taken together, these findings position SRT targeting LKR as a cutting-edge precision metabolic intervention, with broad translational potential extending from rare inborn errors to complex diseases involving oxidative stress and mitochondrial dysfunction.
